# Small GTPase Rab7 is involved in stress adaptation to carbon starvation to ensure the induced cellulase biosynthesis in *Trichoderma reesei*

**DOI:** 10.1186/s13068-024-02504-6

**Published:** 2024-04-20

**Authors:** Lin Liu, Zhixing Wang, Yu Fang, Renfei Yang, Yi Pu, Xiangfeng Meng, Weifeng Liu

**Affiliations:** State Key Laboratory of Microbial Technology, Microbiology Technology Institute, Shandong University, No. 72 Binhai Road, Qingdao, 266237 People’s Republic of China

**Keywords:** Rab GTPase, Rab7, Vesicular transport, Cellulase secretion, *Trichoderma reesei*

## Abstract

**Background:**

The saprophytic filamentous fungus *Trichoderma reesei* represents one of the most prolific cellulase producers. The bulk production of lignocellulolytic enzymes by *T. reesei* not only relies on the efficient transcription of cellulase genes but also their efficient secretion after being translated. However, little attention has been paid to the functional roles of the involved secretory pathway in the high-level production of cellulases in *T. reesei*. Rab GTPases are key regulators in coordinating various vesicle trafficking associated with the eukaryotic secretory pathway. Specifically, Rab7 is a representative GTPase regulating the transition of the early endosome to the late endosome followed by its fusion to the vacuole as well as homotypic vacuole fusion. Although crosstalk between the endosomal/vacuolar pathway and the secretion pathway has been reported, the functional role of Rab7 in cellulase production in *T. reesei* remains unknown.

**Results:**

A TrRab7 was identified and characterized in *T. reesei*. TrRab7 was shown to play important roles in *T. reesei* vegetative growth and vacuole morphology. Whereas knock-down of *Trrab7* significantly compromised the induced production of *T. reesei* cellulases, overexpression of the key transcriptional activator, Xyr1, restored the production of cellulases in the *Trrab7* knock-down strain (P*tcu*-*rab7*^KD^) on glucose, indicating that the observed defective cellulase biosynthesis results from the compromised cellulase gene transcription. Down-regulation of *Trrab7* was also found to make *T. reesei* more sensitive to various stresses including carbon starvation. Interestingly, overexpression of Snf1, a serine/threonine protein kinase known as an energetic sensor, partially restored the cellulase production of P*tcu*-*rab7*^KD^ on Avicel, implicating that TrRab7 is involved in an energetic adaptation to carbon starvation which contributes to the successful cellulase gene expression when *T. reesei* is transferred from glucose to cellulose.

**Conclusions:**

TrRab7 was shown to play important roles in *T. reesei* development and a stress response to carbon starvation resulting from nutrient shift. This adaptation may allow *T. reesei* to successfully initiate the inducing process leading to efficient cellulase production. The present study provides useful insights into the functional involvement of the endosomal/vacuolar pathway in *T. reesei* development and hydrolytic enzyme production.

**Supplementary Information:**

The online version contains supplementary material available at 10.1186/s13068-024-02504-6.

## Background

The filamentous fungus *Trichoderma reesei* (*Hypocrea jecorina*) is one of the prominent cellulase producers in nature [1]. Hyper-cellulolytic *T. reesei* strains have been reported to produce up to 100 g/L lignocellulolytic enzymes [2]. The expression of cellulases in *T. reesei* is first of all stringently controlled at the transcription level by a suite of transcription factors [3–5]. Among them, the Zn_2_Cys_6_ transcription factor Xylanase regulator 1 (Xyr1) has been identified as the master activator for the transcription of cellulase genes in *T. reesei* [6]. In addition to gene transcription, efficient protein secretion involving protein folding in the endoplasmic reticulum (ER), post-translational processing in both the ER and the Golgi as well as vesicle trafficking-mediated delivery of proteins to their proper destination, has been also believed to contribute to the high-level biosynthesis of cellulases in *T. reesei*. However, relatively little is known yet regarding the detailed mechanisms of the secretory pathway in cellulase production in *T. reesei*.

In the conventional protein secretion pathway of eukaryotes, newly synthesized proteins are first translocated into the ER, where proteins undergo folding and preliminary modifications [7]. Properly folded proteins are then packaged into coat protein complex II (COPII) vesicles at ER exit sites and directed to the Golgi wherein proteins are further post-translationally processed (e.g., glycan trimming and extension) [8]. Thereafter, transport vesicles originating from the Golgi deliver various cargo proteins to different destinations, including the organelles of secretory and endocytic pathways, the plasma membrane, and the extracellular space [9, 10]. Therefore, the entire and highly ordered process of post-Golgi vesicle trafficking is finely regulated and coordinated by the Rab GTPases [11], which is the largest subfamily of small G proteins and regulates many trafficking events, including vesicle biogenesis, transport, tethering, docking, and fusion with target membranes [12]. Specifically, fusion of late endosomes or vesicles from TGN with vacuole is mediated by the small GTPase Rab7 and its effectors [13, 14]. Like other Rab GTPases, Rab7 proteins bind GDP in the cytoplasm and remain inactive. Upon the activation by its guanine nucleotide exchange factor (GEF), Rab7 proteins exchange GDP for GTP, which triggers its anchor to the membrane and binding to downstream effectors to regulate vacuole biogenesis and fusion [15]. In yeast, Rab7 is mainly localized in the vacuolar membrane and is necessary for vesicle docking and vacuole-to-vacuole fusion [16]. Similar cellular localization of Rab7 orthologues has been observed in various filamentous fungi and has been shown to play pivotal roles in regulating the fusion of vacuoles and autophagosomes as well as conidiogenesis [17]. Knockdown of Rab7 leads to highly fragmented vacuoles with impaired vegetative growth and stress responses of *Aspergillus nidulans, Fusarium graminearum,* and other filamentous fungi [18, 19]*.* Rab7 has been also reported to play a critical role in the autophagy of *Magnaporthe oryzae* and *F. graminearum* [20, 21]. Specifically, the Δ*Moypt7* mutant displays a defect in the accumulation of autophagic bodies in vacuoles of *M. oryzae* [21]*.*

Vacuoles are the terminal hub of multiple intracellular vesicle trafficking routes and have long been viewed as the recycling center of the cell due to its abundance of decomposing enzymes for macromolecule and even organelle degradation [22, 23]. Recently, however, vacuoles have been reported to play critical roles in nutrient sensing. AMP-activated protein kinase (AMPK) located at the vacuole/lysosome can be activated by the decline of glucose to restore energy homeostasis of the cell [24]. In *Saccharomyces cerevisiae*, Snf1 (sucrose non-fermenting 1 protein kinase), an ortholog of AMPKα, has been also shown to be required for glucose starvation. When the level of glucose declines, Snf1 is activated to inhibit the expression of transcriptional repressors (i.e., the central glucose-responsive repressor Mig1) or to activate transcriptional activators to stimulate the adaption toward carbon starvation conditions [25, 26]. Besides, crosstalk between the endosomal/vacuolar pathway and protein secretion has been suggested. Specifically, the endosomal/vacuolar pathway has been implicated as an alternative route for the delivery of proteins to the cell surface. The endosome has thus been reported to be involved in the extracellular secretion of inulinase InuA in *Aspergillus nidulans* and α-amylase in *Aspergillus oryzae*, respectively [27, 28]. In addition, two chitin synthases, Chs3 and Chs6, were found to accumulate in the lumen of vacuolar compartments of the *Neurospora crassa* hyphae before being delivered to the Spitzenkörper (SPK) of the hyphal apex [29]. The subcellular localization analysis of CBHI-Venus in *T. reesei* also revealed its presence in the vacuoles [30]. Despite these observations and the fact that the fusion of various prevacuolar vesicles with vacuole is mediated by the small GTPase Rab7, the functional roles of Rab7 in *T. reesei* cellulase production remain to be dissected.

In this study, we identified and characterized a Rab7 homolog in *T. reesei*. Knocking down *Trrab7* caused fragmented vacuoles and severe developmental defects in *T. reesei*. Importantly, we provided evidence that TrRab7 is required for *T. reesei* energetic adaptation to carbon starvation to ensure the induced cellulase gene expression. These results revealed that TrRab7 acts as a pivotal regulatory factor in fungal development and cellulase induction in *T. reesei*.

## Results

### Identification and subcellular localization of TrRab7 in *T. reesei*

**Fig. 1:**
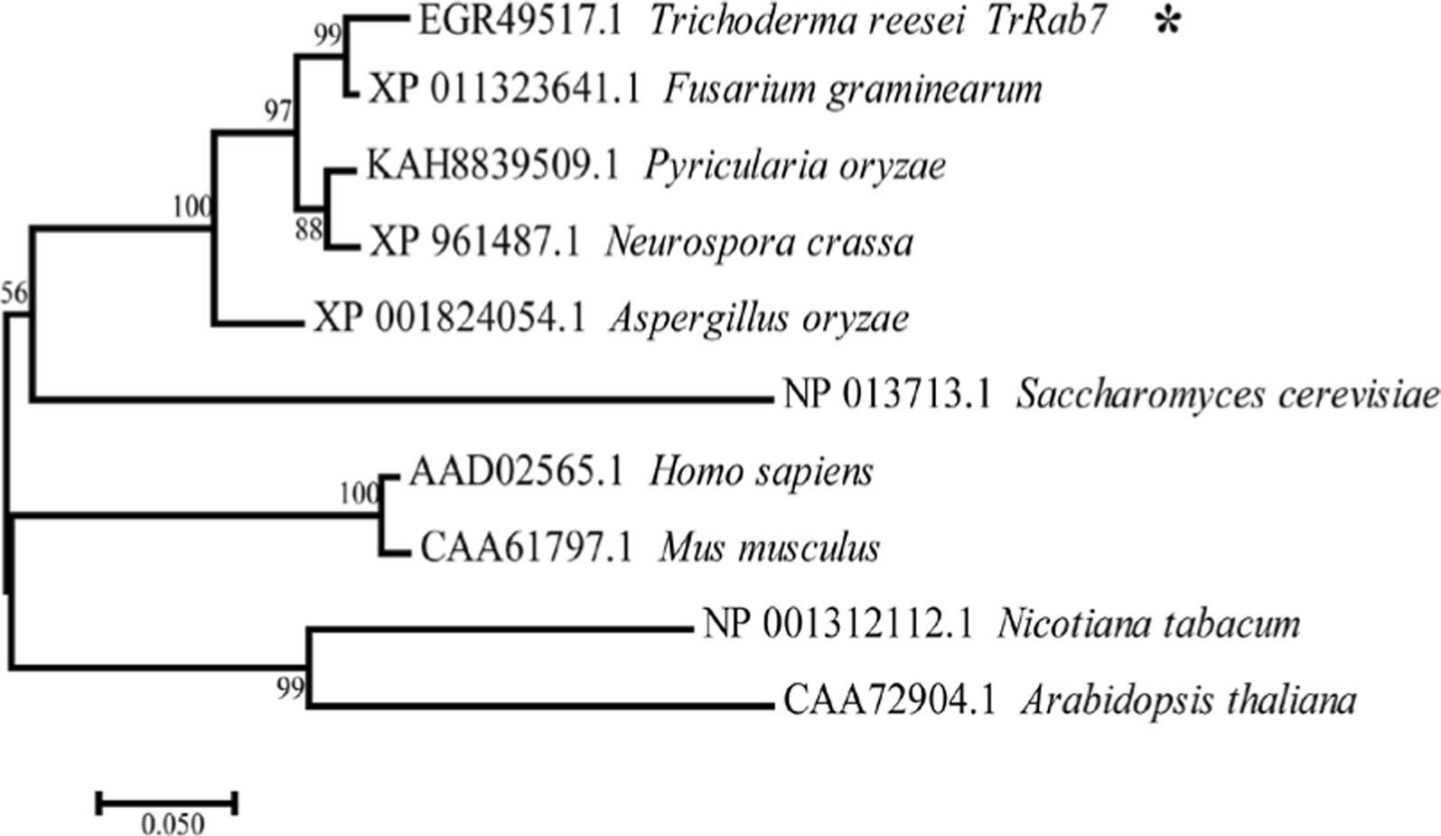
Phylogenetic analysis of TrRab7 and its homologs in different eukaryotic species. Rab7 homologs were well-conserved in filamentous fungi and other eukaryotic taxa. The evolutionary history was inferred using the Neighbor-joining method. The bar corresponds to a genetic distance of 0.050 substitutions per position. The statistical confidence of the inferred phylogenetic relationships was determined by conducting 1000 bootstrap replicates. Each sequence is labeled with their accession number and origin of species

To characterize the function of Rab7 in *T. reesei*, especially its role in the induced biosynthesis of cellulases, a BLAST search was first performed in the *T. reesei* genome database with *S. cerevisiae* Rab7, Ypt7p, which resulted in the identification of Trire2_60331 (TrRab7) as the Rab7 homolog. Phylogenetic analysis revealed that TrRab7 clustered with its corresponding orthologs from various filamentous fungi, indicating a high evolutionary conservation of this small GTPase (Fig. 1). The TrRab7 protein shares a remarkably high sequence identity with its homologs, including FgRab7 of *F. graminearum* (XP_011323641.1, 98% identity), NcRab7 of *N. crassa* (XP_961487.1, 94% identity), AoRab7 of *A. oryzae* (XP_001824054.1, 90% identity), Ypt7p of *S. cerevisiae* (NP_013713.1, 67% identity) and Rab7 of *Homo sapiens* (AAD02565.1, 76% identity). Multiple sequence alignment indicated that all the above proteins possess the signature motifs of Rab GTPases, including the five conserved G-box domains (Rab G1–G5), and a cysteine motif CXC at the carboxyl terminus required for subcellular localization (Additional file 1: Fig. S1).

**Fig. 2:**
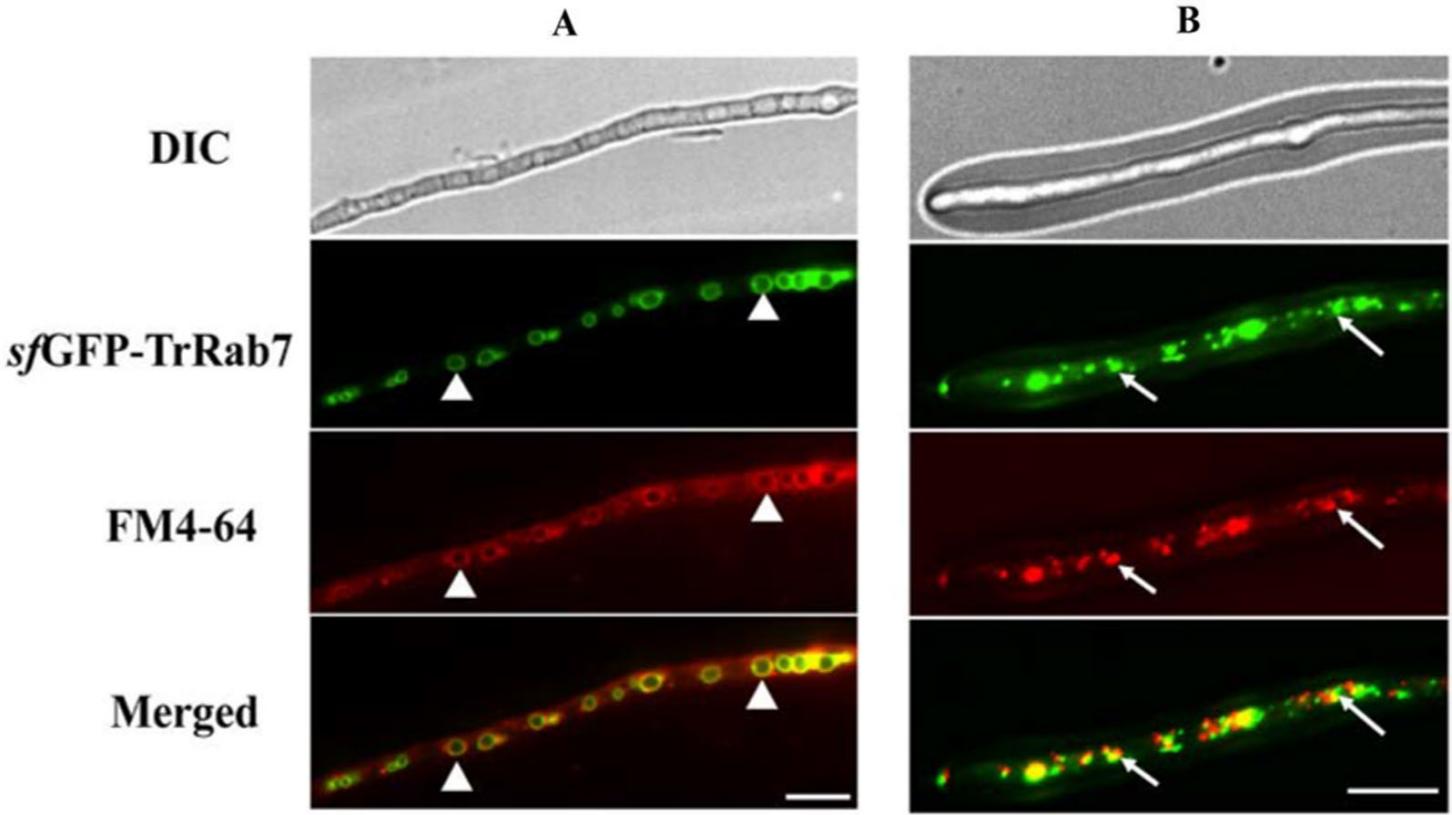
Subcellular localization of *sf*GFP-TrRab7 under cellulase induction condition. **A** Spherical structures (white triangle) observed in fluorescence microscopy analysis of *sf*GFP-TrRab7; **B** Distinct motile punctate structures (arrows) corresponding to pre-vacuolar compartments (PVCs) observed in fluorescence microscopy analysis of *sf*GFP-TrRab7. The strain was grown in MA medium with 1% (w/v) Avicel and mycelia were collected and stained with 10 μM FM4-64 for fluorescence microscopy analysis at 24 h. The fluorescence was examined with a Nikon Eclipse 80i fluorescence microscope. Scale bar: 10 µm. The representative images shown were taken from at least two independent experiments

To determine the subcellular localization of TrRab7 in *T. reesei*, a codon-optimized super folder green fluorescent protein (*sf*GFP) was fused to the N-terminus of TrRab7 and the expression cassette under the control of a copper-controlled *tcu1* promoter was integrated at the *pyr4* locus by homologous recombination (Additional file 1: Fig. S2A) [31, 32]. When no exogenous copper was included in media, *sf*GFP-TrRab7 was expressed. Adding such a *sf*GFP tag did not interfere with the function of TrRab7 in terms of mycelia growth (Additional file 1: Fig. S2B). To localized the vacuole, P*tcu-gfp-Trrab7* was stained by the dye FM4-64, which is taken up by endocytosis and labels the apical vesicle cluster, the putative endosomes, and eventually the vacuolar membrane [33, 34]. Fluorescence analysis of the transformant showed that *sf*GFP-TrRab7 colocalized on the FM4-64 stained membrane of large developed vacuoles with the ring size being about 2 μm in diameter as well as on small punctate structures throughout hyphae (Fig. 2). According to a previous report [35], these ring structures were assumed to be vacuoles with *sf*GFP-TrRab7 being localized to the vacuolar membrane (Fig. 2A). In addition, distinct motile punctate structures corresponding to pre-vacuolar compartments (PVCs) were also observed (Fig. 2B), similar to the localization of their homologs in *N. crassa* [36], *M. oryzae* [37], and *A. nidulans* [38].

### TrRab7 is important for *T. reesei* vacuolar maintenance

**Fig. 3:**
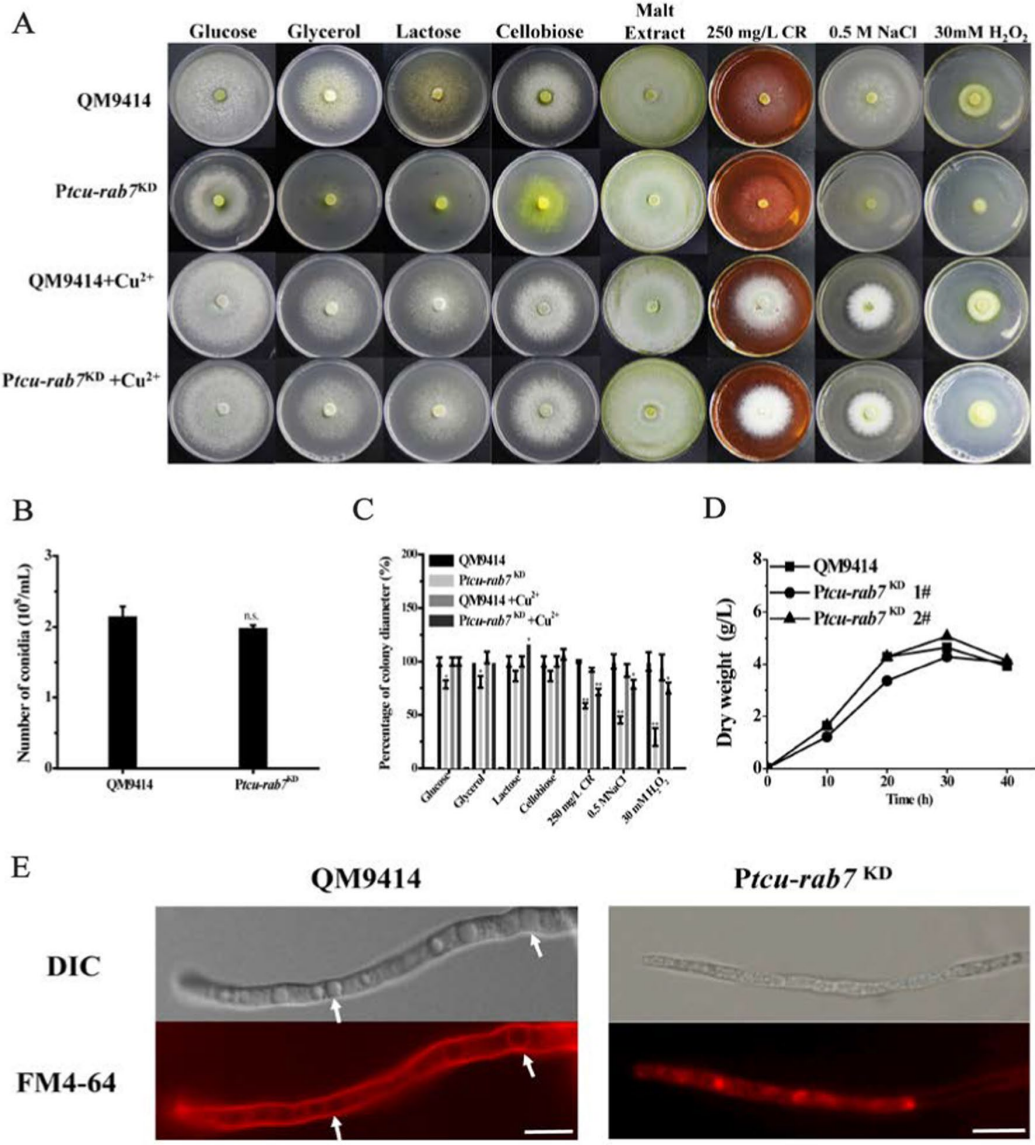
*Trrab7* knock-down influenced the fungal development and vacuolar maintenance of *T. reesei*. **A** Effect of *Trrb7* knock-down on the vegetative growth, cell wall integrity, stress response and conidiation of *T. reesei* strains. Growth of QM9414 and P*tcu*-*rab7*^KD^ strains was examined on plates with various carbon sources (1% w/v) as indicated. The conidiation of QM9414 and P*tcu*-*rab7*^KD^ was performed on malt extract plates. Cell wall integrity was determined on minimal medium (MM) plates containing 250 mg/L Congo red (CR). Osmotic and oxidative stress response tests were performed on minimal medium (MM) plates containing 0.5 M NaCl and 30 mM H_2_O_2_, respectively. QM9414 and P*tcu*-*rab7*^KD^ on different agar plates were kept at 30 °C for 3 days. **B** Number of spores in QM9414 and P*tcu*-*rab7*^KD^ strains on the malt extract was counted by a hemocytometer. **C** Statistical quantitation of percentage in colony diameter of each strain under different agar plates relative to QM9414 cultured with glucose. Significant differences were determined by a two-tailed student's *t* test. *, *P* < 0.05; **P < 0.01; *** *P* < 0.001. **D** Biomass accumulation of the QM9414 and P*tcu*-*rab7*^KD^ in MA medium containing 1% (wt/v) glucose as the sole carbon source. **E** Effect of *Trrab7* knock-down on vacuole formation. QM9414 and P*tcu*-*rab7*^KD^ were grown in MA medium with 1% (w/v) Avicel and mycelia were collected and stained with 10 μM FM4-64 for fluorescence microscopy analysis at 24 h. The fluorescence was examined with a Nikon Eclipse 80i fluorescence microscope. The structure of the normal vacuole in *T. reesei* QM9414 is indicated by arrows in the figure. Scale bar, 10 μm

To investigate the physiological role of TrRab7 in *T. reesei*, *Trrab7* was knocked down by the *tcu1* promoter-controlled RNAi strategy to obtain a conditional *Trrab7* downregulated mutant, P*tcu-rab7*^KD^ (Additional file 1: Fig. S3A). The expressed interference RNA under the control of the *tcu1* promoter in the absence of exogenously added copper would tune down the target mRNA, which was otherwise shut off with inclusion of copper during the culture [39]. When repressed without copper, P*tcu-rab7*^KD^ showed compromised vegetative growth on agar plates with various carbon sources although no difference was observed with conidiation compared with that of QM9414 on malt extract (Fig. 3A, B). In addition, P*tcu-rab7*^KD^ showed reduced growth on plates containing Congo red (CR) compared with QM9414, implicating slightly impaired cell wall integrity. Similarly, P*tcu-rab7*^KD^ showed reduced growth in the presence of 30 mM H_2_O_2_ and 0.5 M NaCl (Fig. 3A, C). In contrast with growth on agar plates, growth and the final biomass of the P*tcu-rab7*^KD^ strain were comparable to that of QM9414 when cultured in a liquid MA medium containing 1% glucose (Fig. 3D). To examine the effect of *Trrab7* knock-down on the intracellular vacuole maturation, hyphae were stained with FM4-64. Microscopy analysis showed that P*tcu-rab7*^KD^ hyphae accumulated a large number of small and fragmented vacuoles, while the wild-type strain formed typical central vacuoles (Fig. 3E). Together, these results showed that TrRab7 plays a role in vacuolar morphogenesis.

### Downregulation of TrRab7 leads to compromised cellulase production in *T. reesei*

**Fig. 4:**
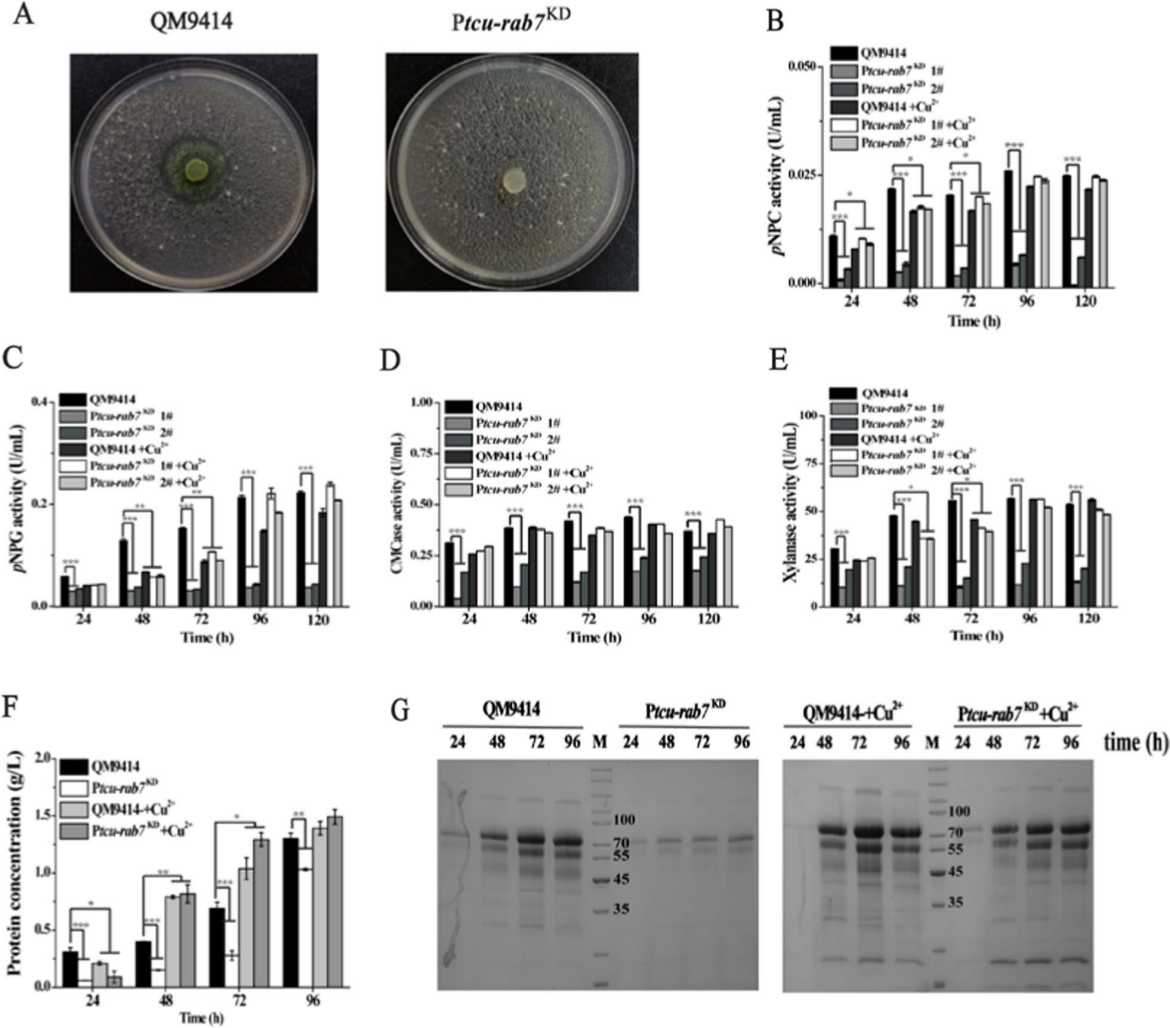
*Trrab7* knock-down compromised the cellulase production of *T. reesei*. **A** Hydrolytic zone formation by the QM9414 and P*tcu*-*rab7*^KD^ strains on MA agar plates covered with a 0.4% (w/v) ground Avicel layer. Extracellular *p*NPC (**B**), *p*NPG (**C**), CMCase (**D**), xylanase (**E**) activities, and protein concentration (**F**) of the culture supernatant of strains cultured using 1% (w/v) Avicel with or without copper for the indicated periods. **G** SDS–PAGE analysis of the culture supernatant of QM9414 and P*tcu*-*rab7*^KD^ with 1% (w/v) Avicel. Culture supernatants (20 μL) were taken at indicated time points and loaded for analysis. M denotes standard molecular weight protein marker. Significant differences were determined by a two-tailed student's *t* test. *, *P* < 0.05; **P < 0.01; *** *P* < 0.001

To examine the role of TrRab7 in the induced biosynthesis of cellulases, the P*tcu-rab7*^KD^ strain was inoculated on an Avicel-containing agar plate. Hardly any hydrolytic zone was observed for the mutant strain (Fig. 4A), indicating that the knock-down of *Trrab7* crippled the cellulolytic activity in *T. reesei.* To verify this result, P*tcu-rab7*^KD^ and QM9414 were cultured in an MA liquid medium with 1% (w/v) Avicel as the sole carbon source, and the extracellular cellulase activities were determined. *Trrab7* knock-down resulted in a dramatic decrease in the extracellular hydrolytic activities, including exo-glucanase (*p*NPCase) (Fig. 4B), β-glucosidase (*p*NPGase) (Fig. 4C), endo-glucanase (CMCase) (Fig. 4D), and xylanase activities (Fig. 4E). In accordance with the reduction in extracellular activities, significantly lower protein concentrations and fewer secreted cellulases were detected for the *Trrab7* downregulated strain compared with QM9414 (Fig. 4F, G). The observed defects were largely recovered when copper was included in the culture to relieve the interference of *Trrab7* expression. To ask whether TrRab7 exerts an effect on the induced transcription of cellulase genes, quantitative reverse transcription PCR (qRT-PCR) revealed that the relative transcription of the two main cellulase genes, *cbh1, eg1,* as well as the transactivator gene *xyr1* was dramatically decreased in the P*tcu*-*rab7*^KD^ strain (Additional file 1: Fig. S3B–D).

Rab GTPases switch between active and inactive states by undergoing a binding exchange between GTP and GDP [21]. To further verify the functional role of TrRab7 in *T. reesei* development and cellulase production, *T. reesei* strains that expressed either a constitutively active (CA) TrRab7 (Q68L) or an inactive dominant negative (DN) TrRab7 (T23N) driven by the *tcu1* promoter at the *pyr4* locus of QM9414 strain were constructed (Additional file 1: Fig. S2A). While neither TrRab7 mutant affected *T. reesei* growth on glucose (Additional file 1: Fig. S2B), TrRab7 (T23N) displayed a drastic decrease in the extracellular *p*NPCase activity (Additional file 1: Fig. S4). This was in sharp contrast with TrRab7 (Q68L), which caused hardly any change in extracellular enzymatic activities compared with that of QM9414, indicating that an active TrRab7 is required for *T. reesei* cellulase production.

### The defective cellulase production in P*tcu-rab7*^KD^ stems from the compromised cellulase gene transcription

**Fig. 5:**
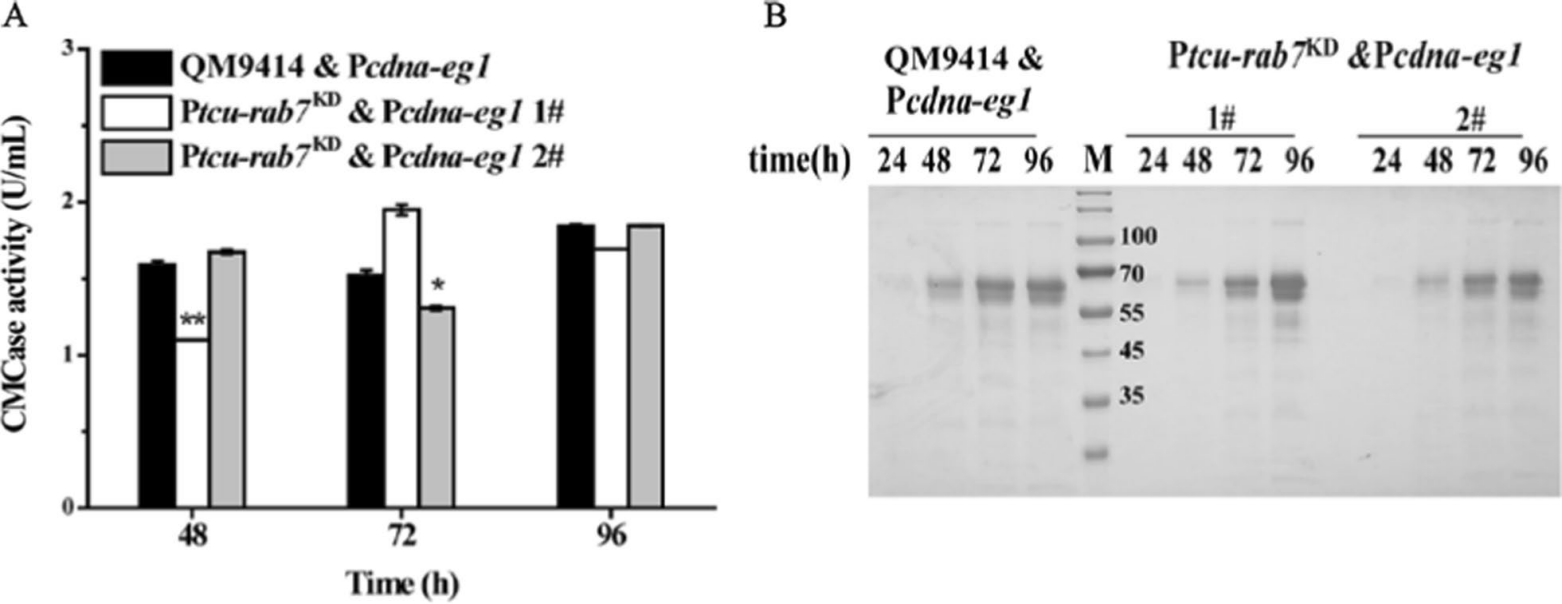
Tr*rab7* knock-down showed no large effect on the secretion of overexpressed EG1. **A** Extracellular CMCase activities of the culture supernatant from QM9414, QM9414 & P*cdna*-*eg1*, and P*tcu*-*rab7*^KD^ & P*cdna*-*eg1* strains cultured with 1% (w/v) glucose at indicated time points. Significant differences were determined by a two-tailed student's *t* test. *, *P* < 0.05; **P < 0.01; ***P < 0.001. **B** SDS–PAGE analysis of the culture supernatants of indicated strains cultured with 1% (w/v) glucose. *M* denotes standard molecular weight protein marker. Culture supernatants (20 μL) were taken at indicated time points and loaded for analysis. As the parental strains QM9414 and P*tcu-rab7*^*KD*^ did not produce significant extracellular proteins with glucose as the sole carbon source, the observed protein band with the highest intensity on SDS–PAGE between 55 and 70 kD was EG1

**Fig. 6:**
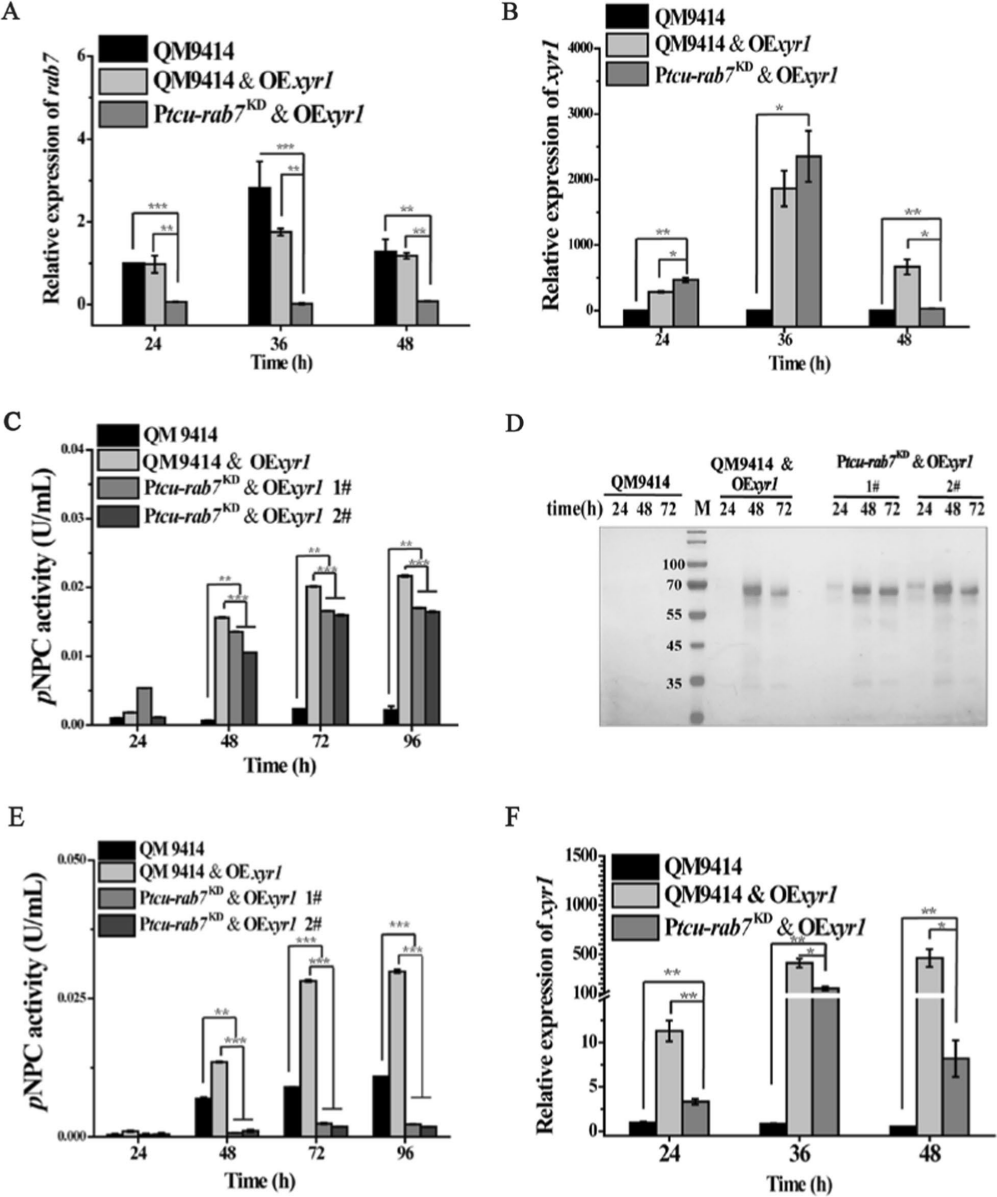
Overexpression of Xyr1 rescued the defect in cellulase production of P*tcu-rab7*^KD^ with glucose but not Avicel. **A**, **B** Quantitative RT-PCR analyses of the relative transcription of *rab7* (**A**) and *xyr1* (**B**) of the QM9414, QM9414 & OE*xyr1,* and P*tcu*-*rab7*^KD^ & OE*xyr1* strains cultured with 1% (w/v) glucose at the indicated time points. **C** Extracellular *p*NPC hydrolytic activities of the culture supernatant from QM9414, QM9414 & OE*xyr1*, and P*tcu*-*rab7*^KD^ & OE*xyr1* strains cultured with 1% (w/v) glucose. **D** SDS–PAGE analysis of the culture supernatants of the indicated strains cultured with 1% (w/v) glucose. Culture supernatants (20 μL) were taken at the indicated time points and loaded for analysis. M denotes standard molecular weight protein marker. **E** Extracellular *p*NPC hydrolytic activities of the culture supernatant of the QM9414, P*tcu*-*rab7*^KD^, QM9414 & OE*xyr1,* and P*tcu*-*rab7*^KD^ & OE*xyr1* strains cultured with 1% (w/v) Avicel at indicated time points. **F** Quantitative RT-PCR analyses of the relative transcription of *xyr1* cultured with 1% (w/v) Avicel at the indicated time points. Significant differences were determined by a two-tailed student's *t* test. *, *P* < 0.05; **P < 0.01; ***P < 0.001

To further investigate whether interference of *Trrab7* affects a step downstream of cellulase gene transcription, and to evidence that the reduced cellulase production of P*tcu-rab7*^KD^ is not due to the compromised secretory capability, the major endoglucanase, EG1, was expressed from the constitutive promoter *cdna1* at the *pyr4* locus of the P*tcu*-*rab7*^KD^ strain and QM9414, respectively [40]. Both extracellular CMCase activity and SDS–PAGE analyses showed that EG1 produced in P*tcu*-*rab7*^KD^ was comparable with that in QM9414 & P*cdna*-*eg1* when cultured on glucose (Fig. 5), demonstrating that the secretory pathway of P*tcu*-*rab7*^KD^ was not impaired. Xyr1 overexpression has been reported to lead to a high-level cellulase gene expression even under repressing conditions [32]. Given that transcription of *xyr1* was also compromised in the P*tcu*-*rab7*^KD^ strain which may well result in the defective cellulase gene expression, we next asked whether overexpression of Xyr1 could correct the cellulase induction defect in P*tcu*-*rab7*^KD^. A recombinant strain (P*tcu-rab7*^KD^ & OE*xyr1*) that overexpressed *xyr1* under the control of a constitutively promoter *cdna1* was constructed in the P*tcu-rab7*^KD^ strain (Fig. 6A, B). When cultured on glucose, a repressing condition that hardly any cellulases would be induced, knockdown of *Trrab7* hardly affected the cellulase gene expression as revealed by both extracellular hydrolytic activity and SDS–PAGE analyses compared with control QM9414 & OE*xyr1* strain (Fig. 6C, D). In contrast, similar restoration was not observed when the Xyr1 overexpressing P*tcu-rab7*^KD^ strain was cultured with Avicel (Fig. 6E, F). Overall, these results indicate that the compromised cellulase production of P*tcu-rab7*^KD^ resulted from the impaired cellulase gene transcription and Xyr1 is a potential regulatory target of TrRab7.

### TrRab7 is involved in stress response and adaptation to carbon starvation

**Fig. 7:**
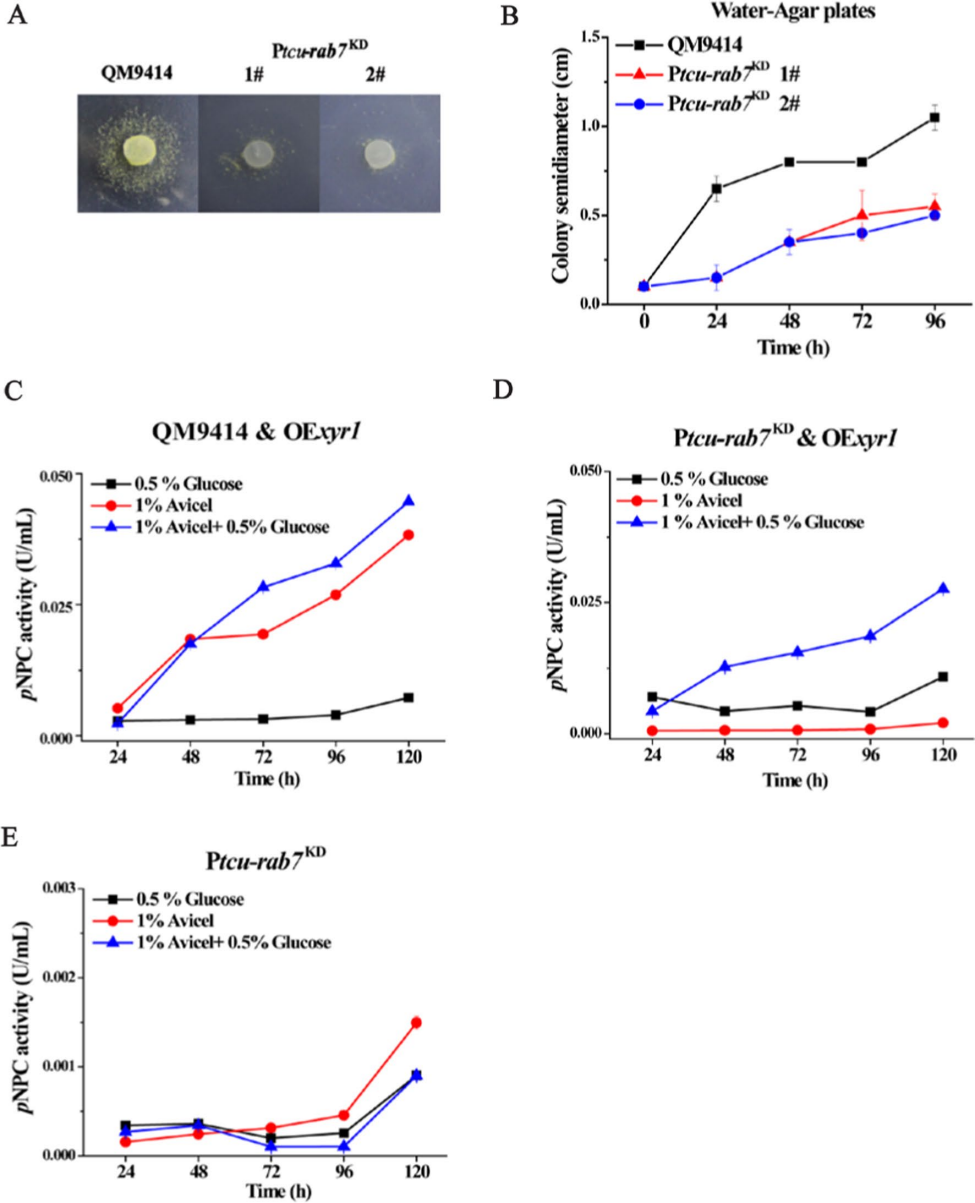
TrRab7 is involved in energetic adaptation and stress response to carbon starvation. **A** The growth of QM9414 and P*tcu*-*rab7*^KD^ on WA plates. **B** Semidiameters determination of QM9414 and P*tcu*-*rab7*^KD^ colonies on WA plates. QM9414 and P*tcu*-*rab7*^KD^ hyphal plugs from MM with 1% glucose plates were placed onto WA plates and incubated at 30 °C for 4 days. Colony diameters were measured daily. Extracellular *p*NPC hydrolytic activities of the supernatant from QM9414 & OE*xyr1* (**C**), P*tcu*-*rab7*^KD^ & OE*xyr1* (**D**), and P*tcu*-*rab7*^KD^ (**E**) strains cultured on 1% (w/v) glucose, 1% (w/v) Avicel, and mixed carbon sources containing 1% (w/v) Avicel and 0.5% (w/v) glucose, respectively

Considering that Xyr1 overexpression failed to recover cellulase production in P*tcu*-*rab7*^KD^ when cultured on Avicel but not on glucose, we speculated that interference of *Trrab7* might make *T. reesei* incapable of adapting to the shift in carbon source and thus suffer from a resultant starvation stress [41, 42]. Such a failure to cope with carbon starvation upon a change from a readily assimilated carbon source to a recalcitrant one may block the initiation of cellulase gene transcription and further aggravate its growth on cellulase-inducing carbon sources like Avicel. To test this hypothesis, hyphal plugs of the *Trrab7* knock-down mutant and QM9414 were transferred from nutrient-rich medium (MM) plates to starvation plates containing only water and agar (WA) to examine their adaptive growth (Fig. 7A, B) [43]. The results showed that QM9414 mycelia were able to expand radially from the initial inoculation site, while the growth of P*tcu*-*rab7*^KD^ was significantly retarded under starvation conditions.

To test whether a relief from the carbon starvation would enable the P*tcu*-*rab7*^KD^ & OE*xyr1* to recover cellulase production on Avicel, the P*tcu*-*rab7*^KD^ & OE*xyr1* and the QM9414 & OE*xyr1* strains were, respectively, cultured on mixed carbon sources (1% Avicel and 0.5% glucose) to see whether including a small amount of glucose could help P*tcu*-*rab7*^KD^ & OE*xyr1* overcome the initial failure of adaptation and successfully initiate the following cellulase induction. While both strains cultured on 0.5% glucose alone produced marginal extracellular *p*NPC hydrolytic activities, including such an amount of glucose with Avicel resulted in a significant cellulase production in P*tcu*-*rab7*^KD^ & OE*xyr1* (Fig. 7C, D). The observed recovery of extracellular hydrolytic activities on mixed carbon sources did not occur with P*tcu*-*rab7*^KD^ (Fig. 7E). Altogether, these results implicate that the defect in cellulase production resulting from *Trrab7* knock-down may be partially caused by the impaired response to carbon starvation stresses.

### Overexpression of Snf1 partially rescued the cellulase production defect of P*tcu-rab7*^KD^ on Avicel

**Fig. 8:**
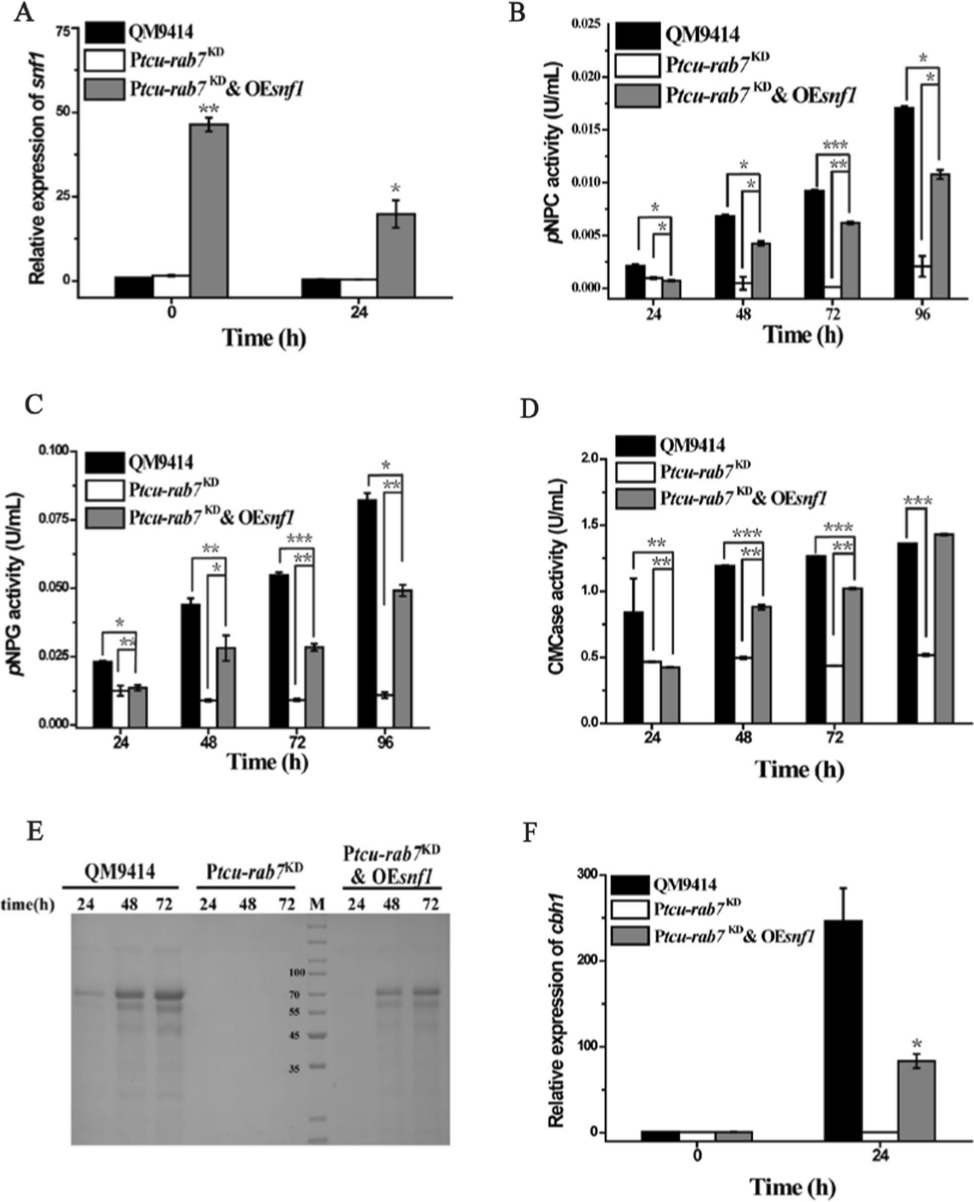
Overexpression of Snf1 partially rescued the cellulase production defect of P*tcu-rab7*^KD^ on Avicel. **A** Quantitative RT-PCR analyses of the relative transcription of *snf1* of QM9414, P*tcu*-*rab7*^KD^, and P*tcu*-*rab7*^KD^ & OE*snf1* strains after induction with 1% (w/v) Avicel. Extracellular *p*NPC (**B**), *p*NPG (**C**), and CMCase activities (**D**) of the culture supernatant from QM9414, P*tcu*-*rab7*^KD^, and P*tcu*-*rab7*^KD^ & OE*snf1* strains cultured with 1% (w/v) Avicel at the indicated points. **E** SDS–PAGE analysis of the culture supernatants of QM9414, P*tcu*-*rab7*^KD^, and P*tcu*-*rab7*^KD^ & OE*snf1* strains cultured with 1% (w/v) Avicel. Equal amounts (20 μL) of culture supernatant were loaded for all strains. M denotes standard molecular weight protein marker. **F** Quantitative RT-PCR analyses of the relative transcription of *cbh1 after* induction with 1% (w/v) Avicel. Significant differences were determined by a two-tailed student's *t* test. *, *P* < 0.05; **P < 0.01; *** *P* < 0.001

Snf1 is an evolutionarily conserved AMP-activated protein kinase (AMPK) that enables exquisite responsivity and control of cellular energetic homeostasis [44]. More recent work has shown that lysosomal AMPK as a primary glucose sensor and glucose deprivation is able to activate lysosomal AMPK independent of gross changes in cellular ATP levels [24, 45]. Given that P*tcu*-*rab7*^KD^ displayed a deficiency in stress response and adaptation to carbon starvation, we explored whether the observed cellulase induction defect of P*tcu*-*rab7*^KD^ could be rescued by overexpression of Snf1. As shown in Fig. 8A–E, overexpression of Snf1 partially restored cellulase production in P*tcu*-*rab7*^KD^ when Avicel was used as the sole carbon source. Correspondingly, the transcription of *cbh1* was shown to be significantly up-regulated in the P*tcu*-*rab7*^KD^ & OE*snf1* strain at 24 h compared to that of P*tcu*-*rab7*^KD^ albeit still lower than that of QM9414 (Fig. 8F). These results thus indicate that TrRab7 is probably involved in *T. reesei* stress response and adaptation to carbon starvation, which constitutes an important step toward the successful initiation of the induced cellulase gene expression.

## Discussion

The filamentous fungus *T. reesei* is well known for its high capacity to secrete large amounts of (hemi)cellulases [46]. Dissecting the molecular mechanism of the efficient cellulase secretion by *T. reesei* is crucial for developing it into a protein cell factory [47]. Whereas the anterograde trafficking of vesicles containing protein cargoes derived from the Golgi to the plasma membrane has been deemed to be responsible for extracellular protein secretion, the endosomal/vacuolar pathway has been reported to affect this process although the exact mechanism remains largely unknown [48, 49]. On the other hand, small Rab GTPases represent crucial regulators coordinating almost all vesicle transport associated with post-Golgi trafficking pathways [50]. In the present study, we identified and elucidated the physiological role of *T. reesei* GTPase Rab7, TrRab7, and its functional involvement in the induced cellulase production. Similar to what has been observed in other filamentous fungi including *A. nidulans* and *F. graminearum* [18, 19], knock-down of *Trrab7* led to highly fragmented vacuoles and impaired the stress responses and vegetative growth of *T. reesei*, indicating that TrRab7 indeed plays an important role in the post-Golgi vesicle fusion with vacuole and strain development in *T. reesei.*

Knock-down of *Trrab7* was further demonstrated to significantly compromise the cellulase production in *T. reesei*. Similar to our result, the endosomal/vacuolar pathway has also been found to be involved in the glycoside hydrolase production in various filamentous fungi. For example, Hernández-González et al. reported that disrupting the fusion of vesicles with early endosomes in *A. nidulans* impaired the extracellular secretion of inulinase InuA, while the morphology and function of the TGN apparatus remained unaffected [27]. In addition, *Aohok1* deletion eliminating the movement of endosome on microtubule (MT) in *A. oryzae* impaired the formation of the apical secretory vesicle cluster, leading to the reduction of the major secretory protein α-amylase, although the distribution of the ER and the Golgi was not affected [51]. These studies together suggest that in addition to the canonical TGN-to-plasma membrane secretory pathway, the endosomal pathway may also be involved in the production of extracellular proteins. However, our further analysis revealed that the compromised cellulase production of the *Trrab7* knock-down strain occurred at the transcriptional level but not at the secretion level. This observation is supported by two lines of evidence. Firstly, hardly any difference was observed in the constitutive expression level of EG1 between P*tcu*-*rab7*^KD^ and the wild-type strain. Secondly, overexpression of Xyr1, the key cellulase gene transcription activator, restored the production of cellulases in P*tcu*-*rab7*^KD^ when cultured on glucose. The downregulation of cellulase gene transcription by tuning down *Trrab7* is in contrast with what was observed for the AP-3 complex subunit in *N. crassa* [52], which mediated the direct fusion of TGN vesicles with vacuole. Loss of AP-3, maintained the transcriptional abundance of major lignocellulose genes at a relatively higher level even at the late stage of induction, thus leading to a significant increase in cellulase production [52]. The different phenotypes as displayed by *Trrab7* and *Ncap-3* may lie in the fact that AP-3-mediated vesicle biogenesis and the following fusion with vacuole represent only one branch of sources to vacuole, while Rab7 participates in regulating all the final vesicle fusions with vacuole.

The efficient cellulase biosynthesis in cellulolytic microbes has been suggested to involve two stages of induction, i.e. no-carbon mimicry and induction [41]. At the no-carbon mimicry stage, a suite of cellulases is slightly expressed and used to initially process extracellular insoluble lignocellulose with the generation of inductive molecules [42]. Such a two-stage mode of cellulase induction (de-repressed and inductive phases) has been supported by transcriptomic analyses revealing the similarity of transcriptional profiles between Avicel and no-carbon conditions [42]. *T. reesei* may initially experience a carbon starvation stress when they are transferred to Avicel and adaptation to such a nutrient shift from a readily assimilated carbon source to a recalcitrant one ensures the successful initiation of a cascade of signal transduction leading to cellulase gene expression. We thus speculated that the failure of Xyr1 overexpression to restore the production of cellulases in P*tcu*-*rab7*^KD^ cultured on Avicel but not on glucose may result from the incapability of adapting to such a carbon shift in the absence of TrRab7. Our observations that P*tcu*-*rab7*^KD^ indeed showed significantly reduced growth on starvation plates and that a lower concentration of glucose to relieve the starvation stress restored cellulase production in P*tcu*-*rab7*^KD^ & OE*xyr1* on Avicel support such a note. Altogether these results demonstrated that TrRab7 may be involved in an energetic adaptation to carbon starvation of *T. reesei*, which contributes to the following cellulolytic response.

Glucose sensing/metabolism has been reported to be critical for derepressing cellulase gene expression in *N. crassa* [53]. Interference with Rab7 expression in *T. reesei* in the present study revealed a defect in carbon starvation adaptation and thus may impair its capacity to respond to the recalcitrant substrate and initiate cellulase gene transcription. AMP-activated protein kinase (AMPK) has long been known as a cellular energy sensor activated by the increase in AMP and ADP to ATP ratios [44]. Once activated, AMPK could modulate the metabolism to restore energy homeostasis of the cell. Recent findings showed that lysosomal AMPK can be activated by the falling levels of glucose, independent of gross changes in cellular ATP levels [24, 45]. In *S. cerevisiae*, the vacuole located AMPK complex has been also demonstrated to play a central role in regulating energy sensing and metabolism of carbon sources [25]. Our observation that overexpression of Snf1 in P*tcu*-*rab7*^KD^ partially restored the cellulase production on Avicel, implicates that TrRab7 is involved in an energetic adaptation and stress response to carbon starvation. It is assumed that knock-down of Rab7 impaired vacuole integrity, which may affect the function of vacuole located AMPK kinase TrSnf1, leading to a deficiency in adaptation to recalcitrant substrate conditions and also the following cellulase gene expression. Considering that the overexpression of Snf1 did not fully restore the P*tcu*-*rab7*^KD^ cellulase production on Avicel, we do not rule out possibilities that TrRab7 directly regulates the activation of Snf1 and that TrRab7 also acts on other unknown effectors contributing to cellulase gene expression. Notwithstanding this, our results provide novel insights into the role of TrRab7 in the induced cellulase gene expression in *T. reesei*.

## Conclusions

TrRab7, a Rab GTPase regulating the fusion of late endosome with vacuole, was identified and characterized in *T. reesei*. TrRab7 was shown not only to play important roles in *T. reesei* vegetative growth, stress response, and vacuole morphology, but also to be involved in an energetic adaptation to carbon starvation of *T. reesei* to ensure the initiation of cellulase biosynthesis. The present study provided evidence that TrRab7 contributes to *T. reesei* cellulase production by pre-adapting cells to a no-carbon mimicry condition before *T. reesei* can successfully initiate cellulase gene expression.

## Methods

### Strains and cultivation conditions

*Escherichia coli* DH5α was routinely used for plasmid construction and amplification and was cultured in lysogeny broth with a rotary shaker (200 rpm) at 37 °C. A uridine–auxotrophic derivative strain *T. reesei* QM9414-Δ*pyr4* [54] was used as the parent strain for construction of *Trrab7* knock-down and *xyr1* or *eg1* overexpression strains. All *T. reesei* strains were maintained on malt extract agar (Sigma Aldrich, USA).

For the gene transcription and cellulase production analysis, *T. reesei* strains were pre-cultured in 250 mL Mandels–Andreotti (MA) medium with 1% (v/v) glycerol as the carbon source for 36 h with a rotary shaker (200 rpm) at 30 °C as previously described [55]. Mycelia were harvested by filtration using the G1 funnel and washed twice with MA medium without any carbon source. Equal amounts of mycelia were then transferred to fresh MA medium without peptone containing 1% (w/v) Avicel (Sigma-Aldrich, USA) or other carbon sources as indicated, and the culture was continued for the indicated periods. The medium was supplemented with 10 mM uridine, 250 μM arginine, 120 μg/mL hygromycin B, and 20 nM copper ions when necessary.

### Cloning and sequence analysis of *Trrab7* in* T. reesei*

Amino acid sequences of Rab7 from *T. reesei* and other relevant species were obtained from the JGI (https://genome.jgi.doe.gov/) and NCBI (http://blast.ncbi.nlm.nih.gov/Blast.cgi) databases. Amino acid sequence alignment was performed using ClustalW (https://www.genome.jp/tools-bin/clustalw). The phylogenetic analysis of TrRab7 was performed with MEGA7.0 using the neighbor-joining method with 1000 bootstraps [56].

### Plasmids and strains construction

To knock down *Trrab7* (Tr_60331, jgi|Trire2) using the previously described RNA interference approach [57], the 2.0 kb DNA fragments of upstream and downstream of *asl1* (encoding the argininosuccinate lyase of *T. reesei* [58], protein ID Tr_80268) was amplified from QM9414 genomic DNA and inserted into the *Hind*III/*Pme*I and *EcoR*I/*BamH*I sites of pUC-*pyr4* [59], respectively, to obtain the pUC*-pyr4::asl1* plasmid. DNA fragments corresponding to the *tcu1* promoter (1.7 kb), I_*cel5a*_ intron (179 bp) and *cel6a* (1.0 kb) were amplified from the pKD-hph plasmid [39] and further ligated into pUC*-pyr4::asl1* through a One Step Cloning Kit (Yeasen, China) resulting in the pKD*-pyr4::asl1*-P*tcu1* plasmid. A 174 bp DNA fragment containing the *Trrab7* coding sequence and its reversed complemented sequence was amplified from QM9414 genomic DNA and inserted into *Sgs*I/*Bgl*II and *Spe*I sites of pKD*-pyr4::asl1*-P*tcu1*, respectively, yielding the *Trrab7* knock-down vector pKD*-pyr4::asl1*-P*tcu1*-*rab7*^KD^. The vector was further linearized with *Xag*I and transformed into the QM9414-Δ*pyr4* strain to select its targeted insertion at the *asl1* locus.

To overexpress *eg1* in QM9414-Δ*pyr4* at the *pyr4* locus, the 2.0 kb fragments of upstream and downstream of the *pyr4* gene were amplified from QM9414 genomic DNA and inserted into the *Hind*III/*Pme*I and *EcoR*I/*Nco*I sites of the pMD*-hph* plasmid [59], respectively, to obtain the pMD*-hph::pyr4* plasmid. DNA fragments of the P_*cdna1*_ promoter (0.9 kb), the T_*cel6a*_ terminator (1.0 kb), and the full sequence eg*1* were amplified from QM9414 genomic DNA and further ligated into the *Pme*I/*Asc*I, *Not*1/*Xho*I and *Asc*I/*Not*I of pMD*-hph::pyr4*, respectively, resulting in pMD-P*cdna-eg1*. Similarly, to overexpress *xyr1* in the P*tcu*-*rab7*^KD^ strain, the P_*cdna1*_ promoter (0.9 kb), the T_*cel6a*_ terminator (1.0 kb), and the full ORF of *xyr1* were amplified from QM9414 genomic DNA and further ligated into the *Pme*I/*Asc*I, *Not*1/*Xho*I and *Asc*I/*Not*I of pMD*-hph::pyr4*, respectively, resulting in pMD-OE*xyr1*. The constructed plasmids were transformed into the QM9414-Δ*pyr4* and P*tcu*-*rab7*^KD^ strains to obtain OE*xyr1* and P*cdna-eg1*, respectively.

To determine the subcellular localization of TrRab7, a DNA fragment containing the codon-optimized *sf*GFP-GGGGS coding sequence (commercial gene synthesis from GENEWIZ, China) was fused at the 5’ terminus of *Trrab7* coding sequence. The resulting PCR fragment (*sfgfp*-*Trrab7*) was ligated into the pMD*-hph::pyr4* plasmid, yielding the pMD-P*tcu1*-*gfp*-*rab7* plasmid. To construct the constitutively active (CA, Q68L) and domain negatively (DN, T23N) mutants of TrRab7, the mutated *Trrab7* was obtained by fusion PCR. The generated mutant sequences were PCR-fused with *sf*GFP-GGGGS and then ligated into the pMD*-hph::pyr4* plasmid to obtain pMD-P*tcu1*-*gfp*-*rab7*-Q68L and pMD-P*tcu1*-*gfp*-*rab7*-T23N, respectively. The pMD-P*tcu1*-*gfp*-*rab7*, pMD-P*tcu1*-*gfp*-*rab7*-Q68L, and pMD-P*tcu1*-*gfp*-*rab7*-T23N plasmids were transformed into the *T. reesei* QM9414-Δ*pyr4* strain to obtain P*tcu-gfp-Trrab7*, P*tcu-gfp-Trrab7-Q68L* and P*tcu-gfp-Trrab7-T23N* strain, respectively.

For overexpressing *snf1* in the P*tcu*-*rab7*^KD^ strain at the *pyr4* locus, the *pyr4* gene of the pMD*-hph::pyr4* plasmid was replaced with the *asl* expression cassette to obtain the pMD*-asl::pyr4* plasmid. The full sequence of *snf1* (Tr_45998) was amplified from QM9414 genomic DNA and further ligated into the *Asc*I/*Not*I of pMD*-asl::pyr4*, resulting in pMD-OE*snf1*. The resultant plasmid was transformed into the P*tcu*-*rab7*^KD^ strain to obtain the corresponding *snf1* overexpression strain.

All the plasmids were linearized before transformation. Transformation of *T. reesei* was performed by PEG–CaCl_2_ mediated protoplast transformation as previously described [60]. Transformants were selected on the minimal medium either for uridine or arginine prototroph, or resistance to hygromycin B (120 μg/mL). All the primers used for plasmid construction and transformant verification are listed in Additional file 1: Table S1.

### Vegetable growth and conidiation assays

To compare the vegetative growth on solid media, *T. reesei* strains were pre-cultured on minimal media agar plate for 3 days, and then a slice of agar with the same area of growing mycelia of the corresponding strains (1 cm in diameter) was taken from the plate and inoculated on minimal media agar plates containing different carbon sources (1% glucose, 1% glycerol, 1% lactose, and 1% cellobiose). For conidiation, malt extract agar plates were used and incubated for 5 days. For the examination of hydrolytic zone formation on Avicel, MA agar plates covered with a 0.4% (w/v) ground Avicel layer were used and incubated for 5 days. Similarly, a slice of agar was transferred to the center of water–agarose (WA, 1% agar in Milli-Q water) plates without any other nutrient to analyze the starvation-induced foraging response. WA plates were incubated at 30 ℃ for 4 days and colony diameters were measured daily.

To analyze biomass accumulation in MA liquid medium, *T. reesei* strains were pre-cultured in MA medium containing 1% glycerol for 36 h, and then an equal amount of wet mycelia was transferred to fresh MA medium with 1% (w/v) glucose as the sole carbon source. Cultures were incubated in a rotary shaker (200 rpm) at 30 °C. The mycelia were collected at indicated growth intervals and dried at 80 ℃ for 48 h. The mycelial dry weight was measured.

To test the cell wall integrity of *T. reesei*, strains were inoculated on MM plates with 1% (w/v) glucose and 250 mg/L Congo red. To test *T. reesei* sensitivity to various stress conditions, strains were inoculated on MM plates with 1% (w/v) glucose and 30 mM H_2_O_2_ (oxidative stress), or 0.5 M NaCl (osmotic stress). After incubation at 30 ℃ for 3 days, the mycelia diameter under the indicated conditions was measured.

### Staining and fluorescence microscopy analysis

To visualize the cellular localization of *sf*GFP-TrRab7 and its mutants in live cells, recombinant strains were grown in MA medium containing 1% (w/v) Avicel for 24 h at 30 °C. Mycelia samples were taken for microscopic analysis. Vacuolar membranes were stained with FM4-64 [N(3-triethylammonium-isopropyl)-4-(6-(4-(diethylamino)phenyl)hexatrienyl)pyridinium-dibromide] (Molecular Probes) according to the procedures described by Parton et al. [33, 61]. Fungal hyphae were harvested and suspended in 0.5 mL of MA medium containing 10 μM FM4-64 in dimethyl sulfoxide and then were incubated with shaking (200 rpm) for 10 min at 30 °C. The stained hyphae were washed twice by fresh media without dye. Afterwards, the cultures were kept at 30 °C in fresh media without dye for another 30 min, followed by microscopy analysis.

All samples were imaged with a Nikon Eclipse Ti-E inverted fluorescence microscope (Nikon, Japan) using a 60 × 1.4 NA oil immersion objective (Plan Apo VC). Filter sets for GFP (excitation 450 to 490 nm; emission 510 to 560 nm) and TRITC (excitation 527 to 552 nm; emission 577 to 633 nm). All images were captured and processed using the NIS-ELEMENTSAR software.

### Enzymatic activity and protein analysis

The activities of exo-glucanase (*p*NPCase) and β-glucosidases (*p*NPGase) were determined by measuring the amount of released *p*-nitrophenol using *p*-nitrophenyl-β-d-cellobioside (*p*NPC, Sigma) and *p*-nitrophenyl-β-d-glucopyranoside (*p*NPG, Aladdin) as substrates, respectively. Reaction mixtures containing 50 μL of culture supernatant and 50 μL of corresponding substrates, in 100 μL 50 mM sodium acetate (pH 4.8) were incubated for 30 min at 50 °C. One unit (U) of *p*NPC or *p*NPG hydrolytic activity is defined as the amount of enzyme releasing 1 μmol of *p-*nitro-phenyl per minute.

The activities of CMCases and xylanase were determined using 1% sodium carboxymethyl cellulose (CMC–Na, Sigma) and 0.5% Beechwood xylan (Megazyme) as substrates, respectively. Reaction mixtures containing 60 μL of appropriately diluted culture supernatant and 60 μL of the respective substrates in 50 mM sodium acetate (pH 4.8) were incubated for 30 min at 50 °C. One unit (U) of activity is defined as the amount of enzyme releasing 1 μmol reducing sugar per minute. The reducing sugar released in the mixture was determined by the 3, 5-dinitrosalicylic acid method with glucose as the standard.

Total secreted proteins were determined using the Bradford protein assay with BSA as the standard. Three biological replicates were carried out for each experiment. SDS–PAGE and Western blotting were performed according to standard protocols [62]. Equal amounts of culture supernatant were loaded for SDS–PAGE analysis of the extracellular proteins.

### Nucleic acid isolation and quantitative real-time PCR

Fungal genomic DNA was extracted with a fungal DNA miniprep kit (Omega Biotech, USA) according to the manufacturer’s protocol. The RNA-easy isolation reagent (Vazyme, Nanjing, China) and TURBO DNA-free kit (Amibon, Austin, TX, USA) were used for Total RNA extraction and genomic DNA removal, respectively. Reverse transcription was performed using HiScript Q RT SuperMix (Vazyme, Nanjing, China) for qPCR. Quantitative real-time PCRs (qRT-PCR) were performed using Taq Pro Universal SYBR qPCR Master Mix (Vazyme, China) on a LightCycler 96 (Roche, Switzerland). Data were analyzed using the relative quantitation/comparative threshold cycle (∆∆Ct) method. All the data were normalized to the endogenous gene *actin* as control [63, 64]. Three biological replicates were performed for each experiment. Briefly, ΔCt (control) and ΔCt (mutant) were quantified by subtracting Ct (*actin*) from their corresponding gene Ct. The ratio of mutant/control gene expression changes was determined as 2^−ΔΔCt^.

### Statistical analysis

Statistical analysis was performed using Student’s *t* test analysis. At least two to three biological replicates were performed for each analysis. The results and errors are the mean and SD of these replicates, respectively.

### Supplementary Information

**Additional file 1: Figure S1.** Multiple sequence alignment of Rab7 homologs. The amino acid alignment of the Rab7/Ypt7 of *Arabidopsis thaliana* (AtRab7, CAA72904.1), *Nicotiana tabacum* (NtRab7, NP_001312112.1), *Saccharomyces cerevisiae* (Ypt7, NP_013713.1), *Homo sapiens* (HoRab7, AAD02565.1), *Mus musculus* (MmRab7, CAA61797.1), *Aspergillus oryzae* (AoRab7, XP_001824054.1), *Fusarium graminearum* (FgRab7, XP_011323641.1), *Pyricularia oryzae* (PoRab7, KAH8839509.1), *Neurospora crassa* (NcRab7, XP_961487.1), and *Trichoderma reesei* (TrRab7) was performed using CLUSTALW. The five conserved GTP-binding motifs (G1 to G5) and C-terminal motifs (C) were labeled on top of their amino acid sequence. **Figure S2**. Construction and growth of GFP-TrRab7 and its mutant strains. (A) Schematic representation of the construction of GFP-TrRab7, and its constitutively active (Q68L) and inactive (T23N) mutants strains*.* The expression of GFP-TrRab7 and its mutants was driven by the P*tcu1* promoter; (B) Biomass accumulation of the QM9414, P*tcu-gfp-rab7,* and mutant strains cultured in MA medium containing 1% (wt/v) glucose as the sole carbon source. **Figure S3.** Relative transcriptions of *Trrab7* and cellulase-related genes in *Trrab7* knock-down strains. The relative transcription of *Trrab7* (A), *xyr1* (B), *cbh1* (C), and *eg1* (D) in QM9414 and P*tcu*-*rab7*^KD^ with 1% (w/v) Avicel as the sole carbon source were determined by quantitative RT-PCR analyses. Significant differences were determined by a two-tailed student's *t* test. *, *P* < 0.05; **P < 0.01; ***P < 0.001. Data were analyzed using the relative quantitation/comparative threshold cycle (∆∆Ct) method. All the data were normalized to the endogenous gene *actin* as control. (E) Extracellular *p*NPC hydrolytic activities of supernatants from QM9414, P*tcu-gfp-rab7,* and mutant strains in MA medium containing 1% (w/v) Avicel as the sole carbon source at indicated time points. **Figure S4.** *p*NPC hydrolytic activities of GFP-TrRab7 and its mutant strains. Extracellular *p*NPC hydrolytic activities of supernatants from QM9414, P*tcu-gfp-rab7,* and mutant strains in MA medium containing 1% (w/v) Avicel as the sole carbon source were determined at indicated time points. **Table S1.** Primers used in this study.

## Data Availability

Data generated in this study are included in the published article.

## Abbreviations

EG1: Endo-glucanase 1
Xyr1: Xylanase regulator 1
Snf1: Sucrose non-fermenting 1 protein kinase
*sf*GFP: Superfolder green fluorescence protein
SDS–PAGE: Sodium dodecyl sulfate–polyacrylamide gel electrophoresis
*p*NPC: *p*-Nitrophenyl-β-d-cellobiose
*p*NPG: *p*-Nitrophenyl-β-d-glucopyranoside
CMC: Carboxymethylcellulose
P*tcu*: The promoter of a copper transporter encoding gene *tcu1* in *T. reesei*
