# Supplementary material for: Small GTPase Rab7 is involved in stress adaptation to carbon starvation to ensure the induced cellulase biosynthesis in Trichoderma reesei
Source: Biotechnol Biofuels Bioprod. 2024 Apr 20;17:55. doi: 10.1186/s13068-024-02504-6 (PMC11032611; doi:10.1186/s13068-024-02504-6)
Supplement: Supplementary file 1 — Additional file 1: Figure S1. Multiple sequence alignment of Rab7 homologs. The amino acid alignment of the Rab7/Ypt7 of Arabidopsis thaliana (AtRab7, CAA72904.1), Nicotiana tabacum (NtRab7, NP_001312112.1), Saccharomyces cerevisiae (Ypt7, NP_013713.1), Homo sapiens (HoRab7, AAD02565.1), Mus musculus (MmRab7, CAA61797.1), Aspergillus oryzae (AoRab7, XP_001824054.1), Fusarium graminearum (FgRab7, XP_011323641.1), Pyricularia oryzae (PoRab7, KAH8839509.1), Neurospora crassa (NcRab7, XP_961487.1), and Trichoderma reesei (TrRab7) was performed using CLUSTALW. The five conserved GTP-binding motifs (G1 to G5) and C-terminal motifs (C) were labeled on top of their amino acid sequence. Figure S2. Construction and growth of GFP-TrRab7 and its mutant strains. (A) Schematic representation of the construction of GFP-TrRab7, and its constitutively active (Q68L) and inactive (T23N) mutants strains. The expression of GFP-TrRab7 and its mutants was driven by the Ptcu1 promoter; (B) Biomass accumulation of the QM9414, Ptcu-gfp-rab7, and mutant strains cultured in MA medium containing 1% (wt/v) glucose as the sole carbon source. Figure S3. Relative transcriptions of Trrab7 and cellulase-related genes in Trrab7 knock-down strains. The relative transcription of Trrab7 (A), xyr1 (B), cbh1 (C), and eg1 (D) in QM9414 and Ptcu-rab7KD with 1% (w/v) Avicel as the sole carbon source were determined by quantitative RT-PCR analyses. Significant differences were determined by a two-tailed student's t test. *, P < 0.05; **P < 0.01; ***P < 0.001. Data were analyzed using the relative quantitation/comparative threshold cycle (∆∆Ct) method. All the data were normalized to the endogenous gene actin as control. (E) Extracellular pNPC hydrolytic activities of supernatants from QM9414, Ptcu-gfp-rab7, and mutant strains in MA medium containing 1% (w/v) Avicel as the sole carbon source at indicated time points. Figure S4. pNPC hydrolytic activities of GFP-TrRab7 and its mutant strains. Extracellular pNPC [file 13068_2024_2504_MOESM1_ESM.docx]

**Supplementary Information**

**Small GTPase Rab7 is involved in stress adaptation to carbon starvation to ensure the induced cellulase biosynthesis in *Trichoderma reesei***

Lin Liu, Zhixing Wang, Yu Fang, Renfei Yang, Yi Pu, Xiangfeng Meng*, Weifeng Liu*

State Key Laboratory of Microbial Technology, Microbiology Technology Institute, Shandong University, No. 72 Binhai Road, Qingdao 266237, People’s Republic of China

*Correspondence should be addressed to Xiangfeng Meng (email: x.meng@sdu.edu.cn) and Weifeng Liu (e-mail: weifliu@sdu.edu.cn)


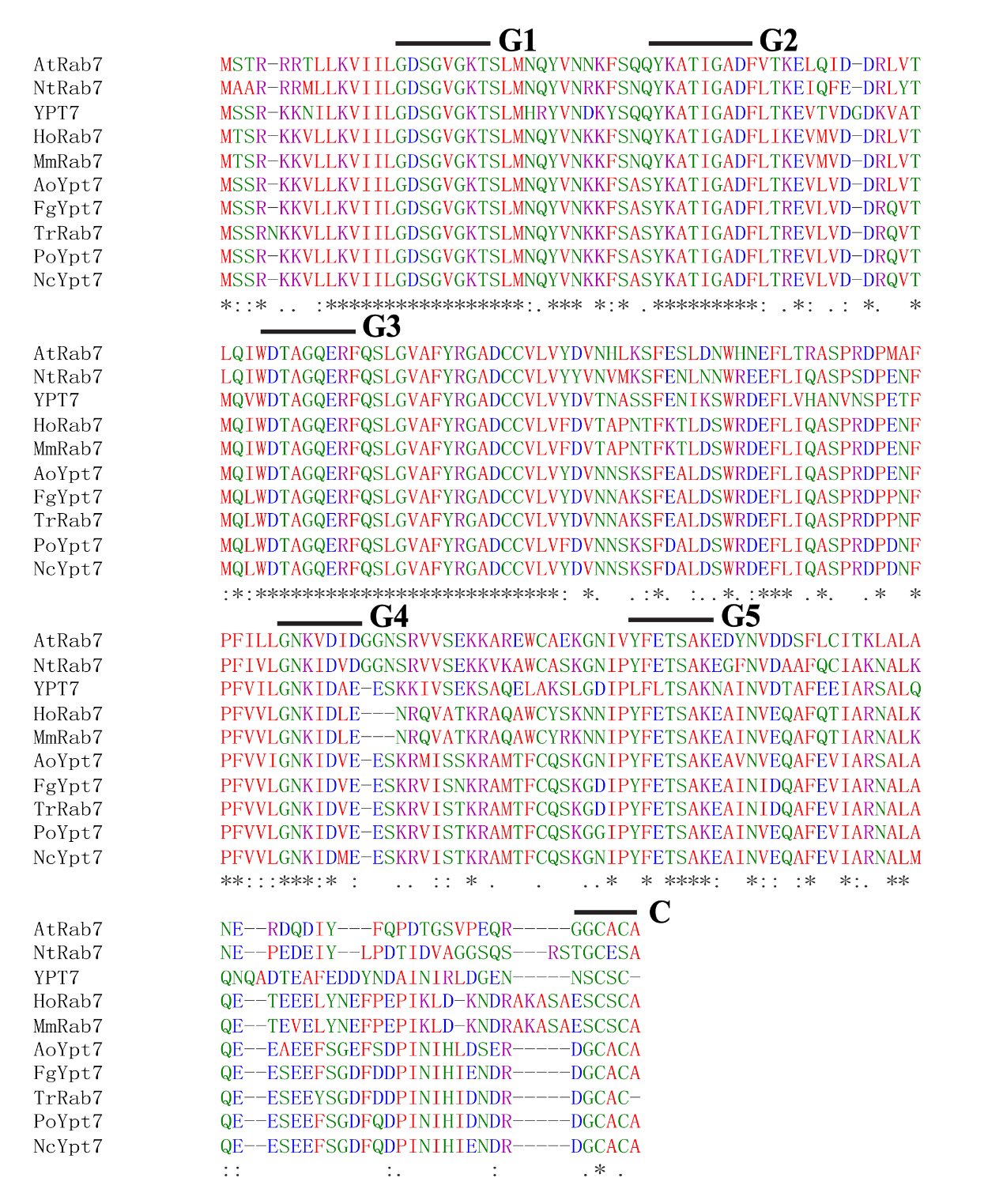


**Fig. S1. Multiple** **sequence alignment of Rab7** **homologs.** The amino acid alignment of the Rab7/Ypt7 of *Arabidopsis thaliana* (AtRab7, CAA72904.1), *Nicotiana tabacum* (NtRab7, NP_001312112.1), *Saccharomyces cerevisiae* (Ypt7, NP_013713.1), *Homo sapiens* (HoRab7, AAD02565.1), *Mus musculus* (MmRab7, CAA61797.1), *Aspergillus oryzae* (AoRab7, XP_001824054.1), *Fusarium graminearum* (FgRab7, XP_011323641.1), *Pyricularia oryzae* (PoRab7, KAH8839509.1), *Neurospora crassa* (NcRab7, XP_961487.1), and *Trichoderma reesei* (TrRab7) was performed using CLUSTALW. The five conserved GTP-binding motifs (G1 to G5) and C-terminal motifs (C) were labeled on top of their amino acid sequence.

**
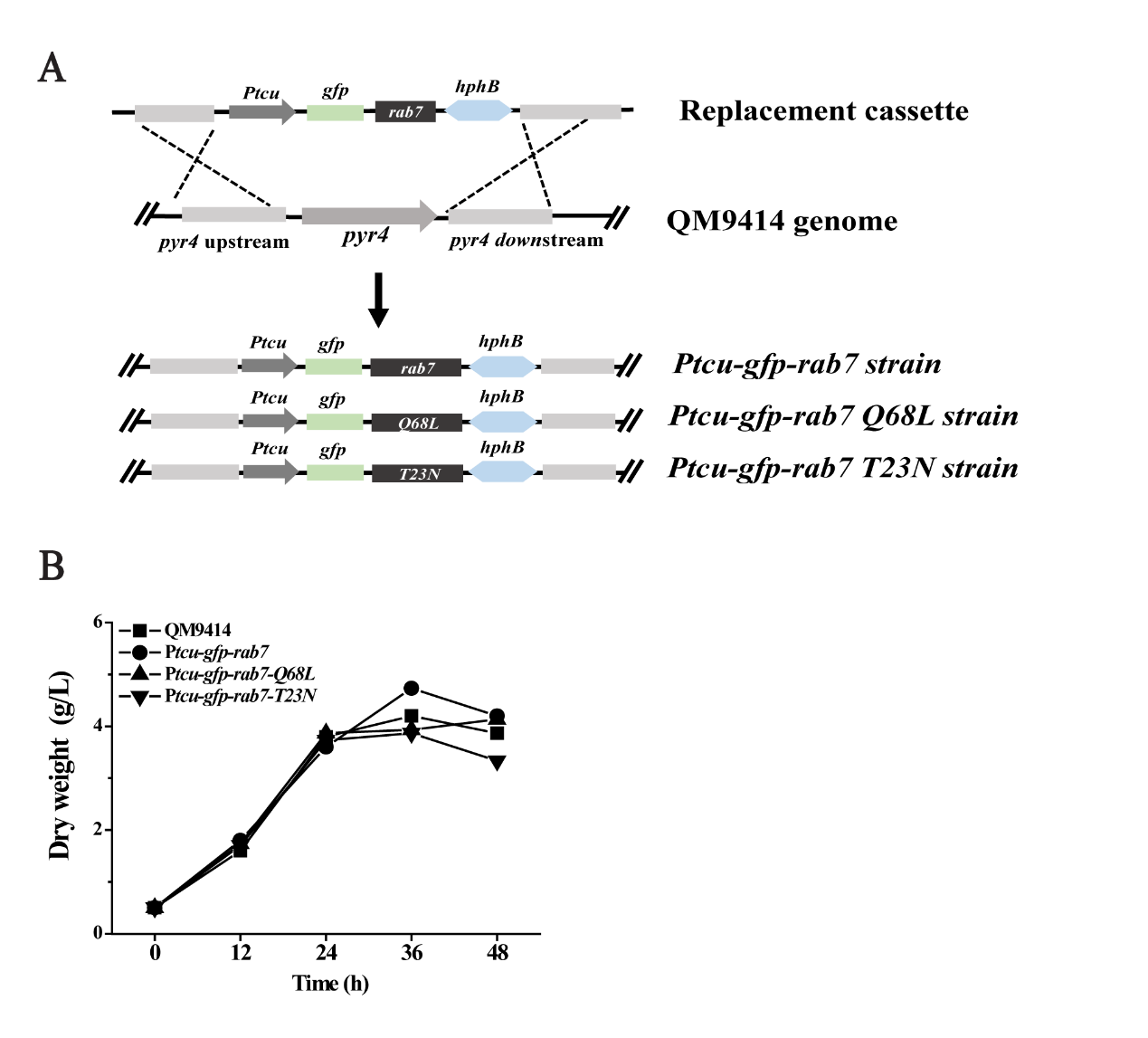
**

**Figure S2**. **The construction and growth of GFP-TrRab7 and its mutant strains.** (A) Schematic representation of the construction of GFP-TrRab7, and its constitutively active (Q68L) and inactive (T23N) mutants strains*.* The expression of GFP-TrRab7 and its mutants was driven by the *tcu1* promoter; (B) Biomass accumulation of the QM9414, P*tcu-gfp-rab7,* and mutant strains cultured in MA medium containing 1% (wt/v) glucose as the sole carbon source.


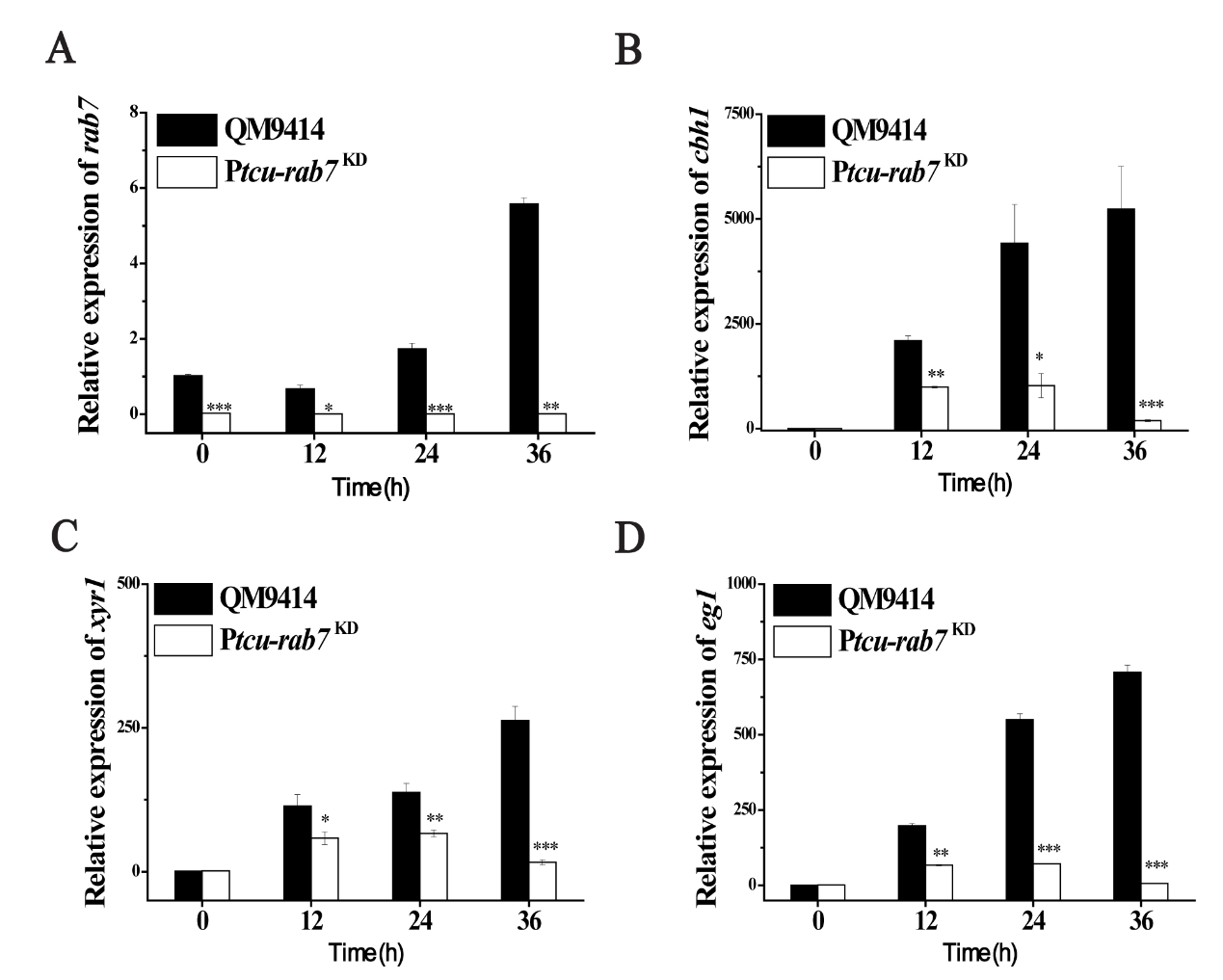


**Figure S3. The relative transcriptions of *Trrab7* and cellulase-related genes in *Trrab7* knock-down strains.** The relative transcription of *Trrab7* (A), *xyr1* (B), *cbh1* (C), and *eg1* (D) in QM9414 and P*tcu*-*rab7*^KD^ with 1% (w/v) Avicel as the sole carbon source were determined by quantitative RT-PCR analyses. Significant differences were determined by a two-tailed student's *t* test. *, P < 0.05; **P<0.01; ***P < 0.001. Data were analyzed by using the relative quantitation/comparative threshold cycle (∆∆Ct) method. All the data were normalized to the endogenous gene *actin* as control.

**
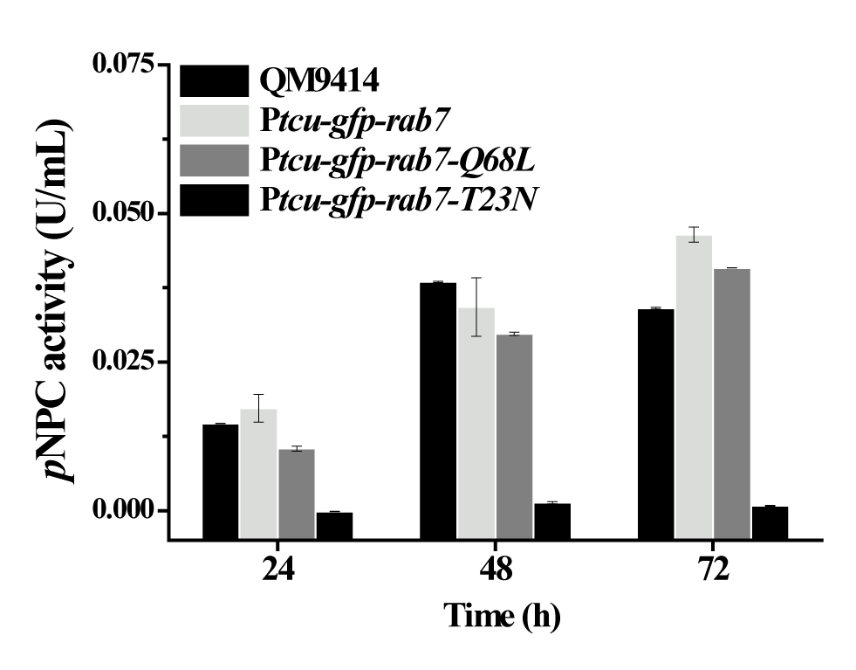
**

**Figure S4. The *p*NPC hydrolytic activities of GFP-TrRab7 and its mutant strains.** Extracellular *p*NPC hydrolytic activities of supernatants from QM9414, P*tcu-gfp-rab7,* and mutant strains in MA medium containing 1% (w/v) Avicel as the sole carbon source were determined at indicated time points.

**Table S1. Primers used in this study**

| **Name** | **Sequence** | **Description** | |
| --- | --- | --- | --- |
| RNAi-*60331*-FS-F | CCTGGTTGATACGACAAGATCT CTGGGATACCGCCGGAC | | Used for plasmid construction for *Trrab7* silencing |
| RNAi-*60331*-FS-R | AACAAGGGTACTCACCCTAGGGGAAAGTTGGGTGGATCT | |  |
| RNAi-*60331*-RCS-F | GTCTTGTTACAGGTTTAAACGGAAAGTTGGGTGGATCTC | |  |
| RNAi-*60331*-RCS-R | CGGTCACGAAAGCCACTAGTCTGGGATACCGCCGGAC | |  |
| P*tcu-*GFP-*rab7*-F | CCTGGTTGATACGACAGATATCATGAGCAAGGGCGAGGAGC | | Used for constructing *sf*GFP-Rab7 fusion protein and its mutant strain. |
| P*tcu-*GFP-*rab7*-R | GCCCGGTCACGAAAGCCACTAGTTTAGCAAGCACATCCGTC | |  |
| GFP-*60331*-F | GTACAAGGGTGGCGGTGGCTCGATGTCTTCGCGCAACAAG | |  |
| GFP-*60331*-R | CTTGTTGCGCGAAGACATCGAGCCACCGCCACCCTTGTAC | |  |
| *60331*-Q68L-F | CTCTGGGATACCGCCGGACTGGAGCGATTCCAGTCGCTG | |  |
| *60331*-Q68L-R | CAGCGACTGGAATCGCTCCAGTCCGGCGGTATCCCAGAG | |  |
| *60331*-T23N-F | GATAGCGGTGTGGGCAAGAACAGCTTGATGAACCAATATG | |  |
| *60331*-T23N-R | CATATTGGTTCATCAAGCTGTTCTTGCCCACACCGCTATC | |  |
| *60331*-N162T-F | CAAAGGAGGCCATCACGATTGACCAAGCATTTG | |  |
| *60331*-N162T-R | CAAATGCTTGGTCAATCGTGATGGCCTCCTTTG | |  |
| P*cdna-xyr1*-F | CCAACAACTTCTCTCGGCGCGCCATGTTGTCCAATCCTCTCC | | Used for plasmid construction for overexpressing *xyr1* or *snf1*. |
| P*cdna-xyr1*-R | GTAACGTTAAGTGGATCGGCGGCCGCTTAGAGGGCCAGACCG | |  |
| P*cdna-snf1*-F | CAACAACTTCTCTCCCATGGATGGCGCGAGCCGAC | |  |
| P*cdna-snf1*-R | ACGTTAAGTCTTAAGGGCGCGCCCATATAGTGTCGT | |  |
| *xyr1*-qF | CCATCAACCTTCTAGACGAC | | Used for quantitative RT-PCR |
| *xyr1*-qR | AACCCTGCAGGAGATAGAC | |  |
| *cbh1*-qF | CTTGGCAACGAGTTCTCTT | |  |
| *cbh1*-qR | TGTTGGTGGGATACTTGCT | |  |
| *actin*-qF | TCCATCATGAAGTGCGAC | |  |
| *actin*-qR | GTAGAAGGAGCAAGAGCAGTG | |  |
| *rab7*-qF | CGTCTTGGTGTACGACGTCA | |  |
| *rab7*-qR | GGTCGAAATCACTCGCTTGC | |  |
